# Supplementary material for: Robust Vaccine-Induced as Well as Hybrid B- and T-Cell Immunity across SARS-CoV-2 Vaccine Platforms in People with HIV
Source: Microbiol Spectr. 2023 May 11;11(3):e01155-23. doi: 10.1128/spectrum.01155-23 (PMC10269828; doi:10.1128/spectrum.01155-23)
Supplement: Supplemental file 1 — Supplemental material. Download spectrum.01155-23-s0001.docx, DOCX file, 0.9 MB [file spectrum.01155-23-s0001.docx]

**SUPPLEMENTARY DATA**

**TO MANUSCRIPT**: “Robust vaccine-induced as well as hybrid B- and T-cell immunity across SARS-CoV-2 vaccine platforms in people with HIV”

**AUTHORS**

Myrthe L. Verburgh^#^, Lisa van Pul^#^, Marloes Grobben^#^, Anders Boyd, Ferdinand W.N.M. Wit, Ad C. van Nuenen, Karel A. van Dort, Khadija Tejjani, Jacqueline van Rijswijk, Margreet Bakker, Lia van der Hoek, Maarten F. Schim van der Loeff, Marc van der Valk, Marit J. van Gils^^^, Neeltje A. Kootstra^^^ and Peter Reiss^^^; for the AGE_h_IV Cohort Study*

# Myrthe L. Verburgh, Lisa van Pul and Marloes Grobben contributed equally to this manuscript

^ Marit J. van Gils, Neeltje A. Kootstra and Peter Reiss contributed equally to this manuscript

* Study group members are listed in the Acknowledgments

**CONTENT**

Figure S1 …………………………………………………………………………….. page 2

Table S1 …………………………………………………………………………….. page 3

Table S2 …………………………………………………………………………….. page 5

Table S3 …………………………………………………………………………….. page 6

Table S4 …………………………………………………………………………….. page 7

Table S5 …………………………………………………………………………….. page 8

Table S6 …………………………………………………………………………….. page 9

Table S7 …………………………………………………………………………….. page 10

Table S8 …………………………………………………………………………….. page 11

Table S9 …………………………………………………………………………….. page 12

Table S10 …………………………………………………………………………….. page 13

Table S11 …………………………………………………………………………….. page 14

Table S12 …………………………………………………………………………….. page 15

Table S13 …………………………………………………………………………….. page 16

Table S14 …………………………………………………………………………….. page 17

Figure S2 …………………………………………………………………………….. page 18

Text S1 …………..………………………………………………………...………..page 19

Figure S3 …………………………………………………………………………….. page 20

**FIGURE S1: SARS-CoV-2 anti-receptor binding domain (RBD) IgG titers pre- and post-vaccination against SARS-CoV-2 in 441 participants of the AGE_h_IV COVID-19 substudy, by HIV-status.**


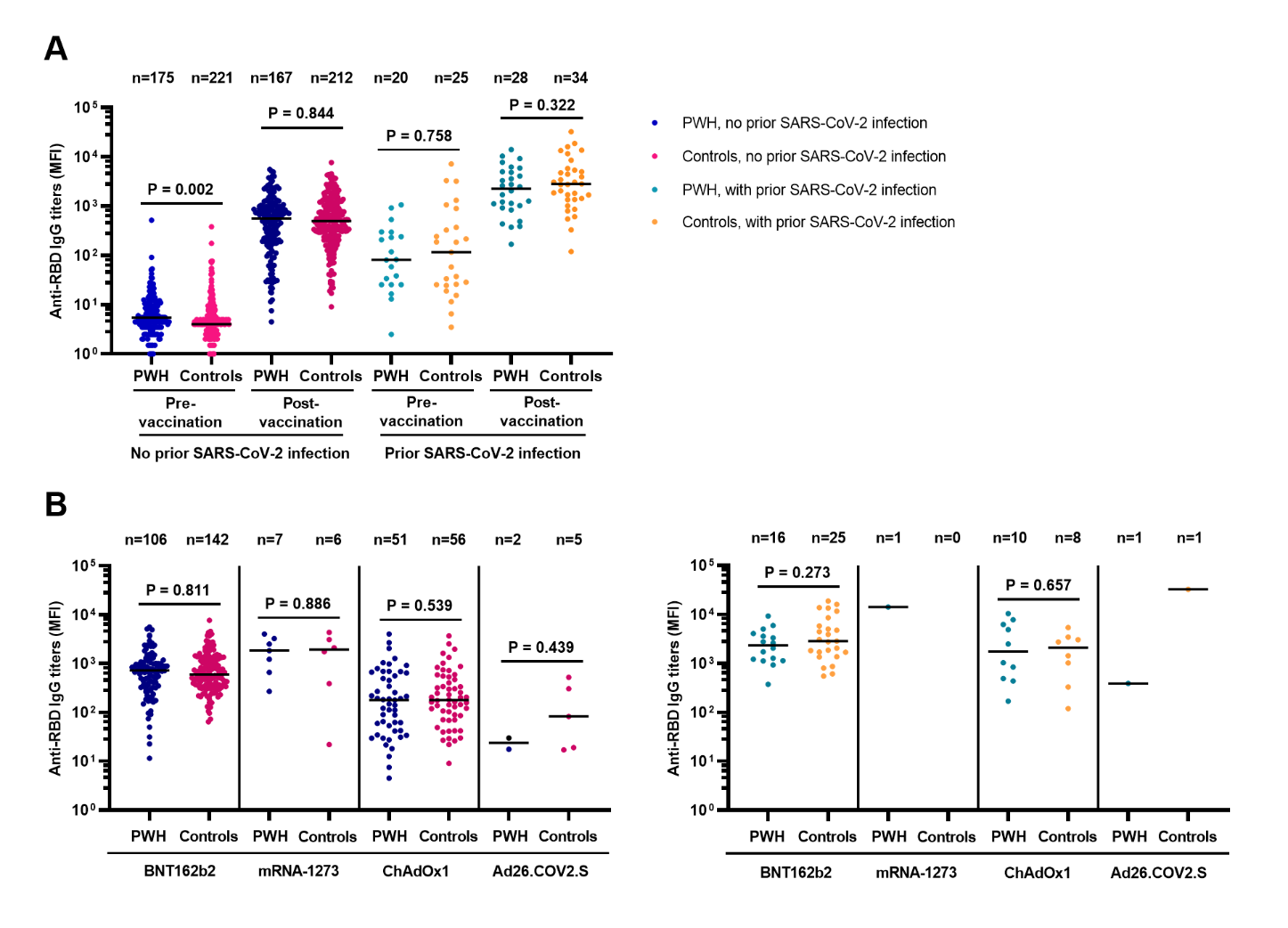


Resulting values are expressed as the Median Fluorescence Intensity (MFI) of at least 50 beads per antigen. P-values, comparing people with HIV (PWH) and controls, were calculated using Wilcoxon rank-sum test. A. Pre- and post-vaccination anti-RBD IgG titers, stratified by prior SARS-CoV-2 infection and HIV-status. B. Post-vaccination anti-RBD IgG titers in those without (left panel) and with (right panel) prior SARS-CoV-2 infection, stratified by vaccine type.

**TABLE S1: Characteristics of 12 anti-spike (S) IgG non-responders, compared to 429 anti-S IgG responders (at 4 to 13 weeks after the last dose of a SARS-CoV-2 vaccine), by HIV-status.**

|  | **People with HIV** | | | **Controls** | | |
| --- | --- | --- | --- | --- | --- | --- |
|  | **Anti-S IgG non-responders (n=6)** | **Anti-S IgG responders (n=189)** | ***P*** | **Anti-S IgG non-responders (n=6)** | **Anti-S IgG responders (n=240)** | ***P*** |
| **Age ^A^** |  |  |  |  |  |  |
| <60 years  60-64 years  65-69 years  ≥70 years | 0 (0.0%)  2 (33.3%)  4 (66.7%)  0 (0.0%) | 66 (34.9%)  47 (24.9%)  37 (19.6%)  39 (20.6%) | .013*** | 1 (16.7%)  4 (66.6%)  1 (16.7%)  0 (0.0%) | 100 (41.7%)  55 (22.9%)  39 (16.2%)  46 (19.2%) | .086*** |
| **Male sex at birth** | 6 (100%) | 178 (94.2%) | >0.999*** | 6 (100%) | 205 (85.4%) | .598*** |
| **Ethnic origin**  Caucasian  African  Asian | 6 (100%)  0 (0.0%)  0 (0.0%) | 184 (97.4%)  5 (2.6%)  0 (0.0%) | >0.999*** | 6 (100%)  0 (0.0%)  0 (0.0%) | 231 (96.5%)  4 (1.7%)  5 (2.1%) | >0.999*** |
| **BMI,** kg/m^2^ **^B^** |  |  | .496*** |  |  | .182*** |
| Underweight (<18.5)  Normal weight (18.5-24.9)  Overweight (25.0-29.9)  Obese (≥30.0) | 0 (0.0%)  4 (66.6%)  1 (16.7%)  1 (16.7%) | 1 (0.5%)  97 (51.3%)  71 (37.6%)  20 (10.6%) |  | 0 (0.0%)  3 (50.0%)  1 (16.7%)  2 (33.3%) | 0 (0.0%)  115 (47.9%)  96 (40.0%)  29 (12.1%) |  |
| **Total comorbidities ^B^**  0 comorbidities  1-2 comorbidities  3-7 comorbidities | 4 (66.7%)  2 (33.3%)  0 (0.0%) | 75 (39.7%)  94 (49.7%)  20 (10.6%) | 0.469*** | 4 (66.7%)  2 (33.3%)  0 (0.0%) | 150 (62.5%)  76 (31.7%)  14 (5.8%) | >0.999*** |
| **SARS-COV-2 vaccine type**  BNT162b2  mRNA-1273  ChAdOx1  Ad26.COV2.S  ChAdOx1 + BNT162b2 | 0 (0.0%)  0 (0.0%)  6 (100%)  0 (0.0%)  0 (0.0%) | 122 (64.6%)  8 (4.2%)  55 (29.1%)  3 (1.6%)  1 (0.5%) | .010*** | 0 (0.0%)  0 (0.0%)  5 (83.3%)  1 (16.7%)  0 (0.0%) | 167 (69.6%)  6 (2.5%)  59 (24.6%)  5 (2.1%)  3 (1.2%) | .002*** |
| **Only one dose of BNT162b2, mRNA-1273 or ChAdOx1 due to prior SARS-CoV-2 infection** | 0 (0.0%) | 2 (1.1%) | >0.999*** | 0 (0.0%) | 8 (3.3%) | .172*** |
| **Days between last vaccine dose and post-vaccination sample ^C^** | 77 (67 – 89) | 64 (45 – 75) | .029* | 80 (74 – 84) | 70 (43 – 77) | .016* |
| **Prior SARS-CoV-2 infection** | 0 (0.0%) | 28 (14.8%) | .596*** | 0 (0.0%) | 34 (14.2%) | >0.999*** |
| **Current CD4 count, cells/mm^3^ ^B^** | 600 (550 – 780) | 650 (500 – 850) | .749* | 965 (530 – 1280) | 810 (650 – 1010) | .585* |
| <350 cells/mm^3^  350-499 cells/mm^3^  500-749 cells/mm^3^  ≥750 cells/mm^3^ | 0 (0.0%)  1 (16.7%)  3 (50.0%)  2 (33.3%) | 19 (10.1%)  25 (13.2%)  74 (39.1%)  71 (37.6%) | >0.999*** | 1 (16.7%)  0 (0.0%)  1 (16.7%)  4 (66.6%) | 2 (0.8%)  21 (8.8%)  71 (29.6%)  146 (60.8%) | .122*** |
| **Current CD8 count, cells/mm^3^ ^B^** | 935 (470 – 1100) | 750 (510 – 990) | .657* | 665 (250 – 1030) | 405 (300 – 560) | .414* |
| <350 cells/mm^3^  350-499 cells/mm^3^  500-749 cells/mm^3^  ≥750 cells/mm^3^ | 1 (16.7%)  1 (16.7%)  0 (0.0%)  4 (66.6%) | 21 (11.1%)  23 (12.2%)  50 (26.5%)  95 (50.2%) | .397*** | 2 (33.3%)  1 (16.7%)  0 (0.0%)  3 (50.0%) | 86 (35.8%)  72 (30.0%)  42 (17.5%)  40 (16.7%) | .225*** |
| **Current CD4/8 ratio ^B^** | 0.72 (0.54 – 1.17) | 0.87 (0.66 – 1.22) | .503* | 1.49 (0.96 – 2.28) | 1.88 (1.34 – 2.56) | .331* |
| < 0.50  0.50 – 0.99  ≥ 1.0 | 1 (16.7%)  3 (50.0%)  2 (33.3%) | 20 (10.6%)  95 (50.2%)  74 (39.1%) | .703*** | 0 (0.0%)  2 (33.3%)  4 (66.7%) | 0 (0.0%)  19 (7.9%)  221 (92.1%) | .085*** |
| **Undetectable HIV-1 viral load ^B^** | 6 (100%) | 187 (99.5%) ^D^ | >0.999*** | NA | NA |  |
| **Time since HIV diagnosis,** yr **^A^** | 20.3 (19.1 – 24.9) | 22.7 (17.0 – 28.0) | .785* | NA | NA |  |
| **Time since first starting ART,** yr **^A^** | 19.2 (14.9 – 21.1) | 20.2 (13.8 – 24.8) | .535* | NA | NA |  |
| **CD4 nadir, cells/mm^3^** | 140 (40 – 170) | 190 (70 – 260) | .187* | NA | NA |  |

Non-response following vaccination against SARS-COV-2 was defined as an anti-S IgG titer <17.8 Median Fluorescence Intensity (MFI) in the post-vaccination sample. All values are n. (%) or median (interquartile range).

A. At moment of post-vaccination blood draw. B. Last available data prior to receiving first vaccine dose of the primary vaccination course. C. Last vaccine dose was either 1) second dose of BNT162b2, mRNA-1273 or ChAdOx1; 2) one dose of Ad26.COV2.S; 3) one dose of BNT162b2, mRNA-1273 or ChAdOx1 in those who only received one vaccine dose. D. HIV viral load was missing in 1/189.

Abbreviations: ART, antiretroviral therapy; BMI, body mass index; NA, not applicable; P, p-value; yr, in years

* Wilcoxon rank-sum test

** Pearson χ2 test

*** Fisher’s exact test

**TABLE S2: Factors associated with SARS-CoV-2 anti-spike IgG titres following vaccination against SARS-COV-2 in 195 people with HIV of the AGE_h_IV COVID-19 substudy.**

|  |  | **Univariable analysis** | | **Multivariable analysis** | |
| --- | --- | --- | --- | --- | --- |
|  | **n/195 (%) ^A^** | **β (95% CI)** | ***P*** | **β (95% CI)** | ***P*** |
| **Age ^B^**  <60 years  60-64 years  65-69 years  ≥70 years | 66 (33.9%)  49 (25.1%)  41 (21.0%)  39 (20.0%) | REF  -0.49 (-0.73 to -0.26)  -0.40 (-0.65 to -0.15)  -0.20 (-0.45 to +0.05) | <.001 | … ^C^ |  |
| **Sex at birth**  Male  Female | 184 (94.4%)  11 (5.6%) | REF  +0.63 (+0.24 to +1.03) | .001 | REF  +0.49 (+0.18 to +0.80) | .002 |
| **Ethnic origin**  Caucasian  African  Asian | 190 (9.4%)  5 (2.6%)  0 (0.0%) | REF  +0.67 (+0.09 to +1.26)  - | .025 | … ^C^ |  |
| **BMI**, kg/m^2^ **^D^**  Underweight (<18.5)  Normal weight (18.5-24.9)  Overweight (25.0-29.9)  Obese (≥30.0) | 1 (0.5%)  101 (51.8%)  72 (36.9%)  21 (10.8%) | -0.11 (-1.41 to +1.19)  REF  +0.15 (-0.05 to +0.35)  -0.23 (-0.54 to +0.08) | .128 | -0.15 (-1.12 to +0.81)  REF  -0.04 (-0.20 to +0.11)  -0.35 (-0.58 to -0.12) | .029 |
| **Total comorbidities ^D^**  0 comorbidities  1-2 comorbidities  3-7 comorbidities | 79 (40.5%)  96 (49.2%)  20 (10.3%) | REF  -0.06 (-0.26 to +0.14)  -0.06 (-0.39 to +0.26) | .828 | … ^C^ |  |
| **Prior SARS-CoV-2 infection**  No  Yes | 167 (85.6%)  28 (14.4%) | REF  +0.73 (+0.48 to +0.97) | <.001 | REF  +0.76 (+0.56 to +0.96) | <.001 |
| **SARS-CoV-2 vaccine**  mRNA-based  vector-based  heterologous ^E^ | 130 (66.7%)  64 (32.8%)  1 (0.5%) | +0.63 (+0.45 to +0.81)  REF  +0.51 (-0.66 to +1.69) | <.001 | +0.67 (+0.52 to +0.83)  REF  +0.72 (-0.24 to +1.68) | <.001 |
| **Days between last vaccine dose and post-vaccination sampling**  *(per 10-day increase)* |  | -0.08 (-0.13 to -0.03) | <.001 | -0.06 (-0.09 to -0.02) | .005 |
| **Current CD4/8 ratio** **^D^**  < 0.50  0.50 – 0.99  ≥ 1.0 | 21 (10.8%)  98 (50.3%)  76 (39.0%) | -0.38 (-0.70 to -0.06)  -0.11 (-0.31 to +0.09)  REF | .006 | -0.45 (-0.69 to -0.21)  -0.11 (-0.26 to +0.04)  REF | .001 |
| **Current CD4 count ^D^**  <350 cells/mm^3^  350-499 cells/mm^3^  500-749 cells/mm^3^  ≥750 cells/mm^3^ | 19 (9.7%)  26 (13.3%)  77 (39.5%)  73 (37.5%) | -0.09 (-0.43 to +0.25)  -0.19 (-0.48 to +0.11)  -0.07 (-0.28 to +0.14)  REF | .665 | … ^C^ |  |
| **Current CD8 count ^D^**  <350 cells/mm^3^  350-499 cells/mm^3^  500-749 cells/mm^3^  ≥750 cells/mm^3^ | 22 (11.3%)  24 (12.3%)  50 (25.6%)  99 (50.8%) | REF  -0.04 (-0.43 to +0.34)  +0.02 (-0.31 to +0.36)  -0.11 (-0.42 to +0.20) | .668 | … ^C^ |  |
| **CD4nadir**  *(per 100 cells/mm^3^ increase)* |  | +0.012 (-0.04 to +0.07) | .669 | … ^C^ |  |
| **Years since HIV diagnosis** **^B^**  *(per 10 years increase)* |  | -0.07 (-0.20 to +0.06) | .289 | … ^C^ |  |
| **Years since starting ART ^B^**  *(per 10 years increase)* |  | +0.009 (-0.14 to +0.16) | .907 | … ^C^ |  |

Values represent regression coefficients (β) of linear regression with 95% confidence interval. Unit is the log_10_ Median Fluorescence Intensity (MFI).

A. Number and percentage of participants for each variable category. B. At moment of post-vaccination blood draw. C. Variable not included in multivariable analysis. D. Last available data prior to receiving first vaccine dose of the primary vaccination course. E. Received one dose of ChAdOx1 and one dose of BNT162b2.

Abbreviations: ART, antiretroviral therapy; BMI, body mass index; CI, confidence interval; P, p-value; REF, reference group.

**TABLE S3: Factors associated with SARS-CoV-2 anti-spike IgG titres following vaccination against SARS-COV-2 in 246 controls without HIV of the AGE_h_IV COVID-19 substudy.**

|  |  | **Univariable analysis** | | **Multivariable analysis** | |
| --- | --- | --- | --- | --- | --- |
|  | **n/246 (%) ^A^** | **β (95% CI)** | ***P*** | **β (95% CI)** | ***P*** |
| **Age ^B^**  <60 years  60-64 years  65-69 years  ≥70 years | 101 (41.0%)  59 (24.0%)  40 (16.3%)  46 (18.7%) | REF  -0.27 (-0.46 to -0.08)  -0.09 (-0.30 to +0.13)  +0.09 (-0.12 to +0.29) | .010 | … ^C^ |  |
| **Sex at birth**  Male  Female | 211 (85.8%)  35 (14.2%) | REF  +0.19 (-0.02 to +0.40) | .079 | REF  +0.16 (+0.003 to +0.32) | .046 |
| **Ethnic origin**  Caucasian  African  Asian | 237 (96.4%)  4 (1.6%)  5 (2.0%) | REF  +0.35 (-0.24 to +0.94)  +0.13 (-0.40 to +0.65) | .461 | … ^C^ |  |
| **BMI**, kg/m^2^ **^D^**  Underweight (<18.5)  Normal weight (18.5-24.9)  Overweight (25.0-29.9)  Obese (≥30.0) | 0 (0.0%)  118 (48.0%)  97 (39.4%)  31 (12.6%) | -  REF  +0.06 (-0.10 to +0.22)  +0.07 (-0.17 to +0.31) | .691 | … ^C^ |  |
| **Total comorbidities ^D^**  0 comorbidities  1-2 comorbidities  3-7 comorbidities | 154 (62.6%)  78 (31.7%)  14 (5.7%) | REF  -0.17 (-0.18 to +0.15)  +0.07 (-0.26 to +0.39) | .892 | … ^C^ |  |
| **Prior SARS-CoV-2 infection**  No  Yes | 212 (86.2%)  34 (13.8%) | REF  +0.78 (+0.59 to +0.97) | <.001 | REF  +0.78 (+0.63 to +0.94) | <.001 |
| **SARS-COV-2 vaccine**  mRNA-based  vector-based  heterologous ^E^ | 173 (70.3%)  70 (28.4%)  3 (1.2%) | +0.59 (+0.44 to +0.74)  REF  +0.54 (-0.08 to +1.16) | <.001 | +0.45 (+0.33 to +0.58)  REF  +0.44 (-0.07 to +0.94) | <.001 |
| **Days between last vaccine dose and post-vaccination sampling**  *(per 10-day increase)* |  | -0.12 (-0.16 to -0.08) | <.001 | -0.09 (-0.12 to -0.06) | <.001 |
| **Current CD4/8 ratio** **^D^**  < 0.50  0.50 – 0.99  ≥ 1.0 | 0 (0.0%)  21 (8.5%)  225 (91.5%) | -  -0.31 (-0.57 to -0.04)  REF | .022 | -  -0.19 (-0.39 to 0.000)  REF | .050 |
| **Current CD4 count ^D^**  <350 cells/mm^3^  350-499 cells/mm^3^  500-749 cells/mm^3^  ≥750 cells/mm^3^ | 3 (1.2%)  21 (8.5%)  72 (29.3%)  150 (61.0%) | -0.62 (-1.29 to +0.06)  -0.13 (-0.40 to +0.14)  -0.16 (-0.33 to +0.002)  REF | .082 | … ^C^ |  |
| **Current CD8 count ^D^**  <350 cells/mm^3^  350-499 cells/mm^3^  500-749 cells/mm^3^  ≥750 cells/mm^3^ | 88 (35.8%)  73 (29.6%)  42 (17.1%)  43 (17.5%) | REF  -0.05 (-0.23 to +0.14)  +0.03 (-0.19 to +0.25)  -0.14 (-0.36 to +0.08) | .536 | … ^C^ |  |

Values represent regression coefficients (β) of linear regression with 95% confidence interval. Unit is the log_10_ Median Fluorescence Intensity (MFI).

A. Number and percentage of participants for each variable category. B. At moment of post-vaccination blood draw. C. Variable not included in multivariable analysis. D. Last available data prior to receiving first vaccine dose of the primary vaccination course. E. Received one dose of ChAdOx1 and one dose of BNT162b2.

Abbreviations: BMI, body mass index; CI, confidence interval; P, p-value; REF, reference group.

**TABLE S4: Factors associated with SARS-CoV-2 anti-receptor binding domain IgG titres following vaccination against SARS-COV-2 in 441 participants of the AGE_h_IV COVID-19 substudy.**

|  |  | **Univariable analysis** | | **Multivariable analysis** | |
| --- | --- | --- | --- | --- | --- |
|  | **n/441 (%) ^A^** | **β (95% CI)** | ***P*** | **β (95% CI)** | ***P*** |
| **HIV status**  Negative  Positive | 246 (55.8%)  195 (44.2%) | REF  -0.06 (-0.18 to +0.06) | .306 | REF  +0.07 (-0.04 to +0.17) | .218 |
| **Age ^B^**  <60 years  60-64 years  65-69 years  ≥70 years | 167 (37.9%)  108 (24.5%)  81 (18.3%)  85 (19.3%) | REF  -0.27 (-0.42 to -0.12)  -0.22 (-0.38 to -0.05)  -0.02 (-0.18 to +0.14) | .001 | … ^C^ |  |
| **Sex at birth**  Male  Female | 395 (89.6%)  46 (10.4%) | REF  +0.29 (+0.10 to +0.48) | .003 | REF  +0.22 (+0.07 to +0.37) | .004 |
| **Ethnic origin**  Caucasian  African  Asian | 427 (96.8%)  9 (2.1%)  5 (1.1%) | REF  +0.41 (-0.003 to +0.82)  +0.31 (-0.23 to +0.86) | .083 | … ^C^ |  |
| **BMI**, kg/m^2^ **^D^**  Underweight (<18.5)  Normal weight (18.5-24.9)  Overweight (25.0-29.9)  Obese (≥30.0) | 1 (0.2%)  219 (49.7%)  169 (38.3%)  52 (11.8%) | -0.19 (-1.41 to +1.04)  REF  +0.09 (-0.03 to +0.22)  +0.002 (-0.19 to +0.19) | .500 | … ^C^ |  |
| **Total comorbidities ^D^**  0 comorbidities  1-2 comorbidities  3-7 comorbidities | 233 (52.8%)  174 (39.5%)  34 (7.7%) | REF  -0.03 (-0.15 to +0.09)  -0.06 (-0.28 to +0.17) | .811 | … ^C^ |  |
| **Prior SARS-CoV-2 infection**  No  Yes | 379 (85.9%)  62 (14.1%) | REF  +0.74 (+0.59 to +0.90) | <.001 | REF  +0.76 (+0.64 to +0.89) | <.001 |
| **SARS-COV-2 vaccine**  mRNA-based  vector-based  heterologous ^E^ | 303 (68.7%)  134 (30.4%)  4 (0.9%) | +0.54 (+0.43 to +0.66)  REF  +0.47 (-0.10 to +1.04) | <.001 | +0.48 (+0.38 to +0.58)  REF  +0.46 (-0.01 to +0.93) | <.001 |
| **Days between last vaccine dose and post-vaccination sampling**  *(per 10-day increase)* |  | -0.11 (-0.14 to -0.08) | <.001 | -0.08 (-0.11 to -0.06) | <.001 |
| **Current CD4/8 ratio** **^D^**  < 0.50  0.50 – 0.99  ≥ 1.0 | 21 (4.8%)  119 (27.0%)  301 (68.3%) | -0.38 (-0.66 to -0.11)  -0.13 (-0.26 to -0.003)  REF | .006 | -0.47 (-0.70 + -0.25)  -0.13 (-0.25 to -0.009)  REF | <.001 |
| **Current CD4 count ^D^**  <350 cells/mm^3^  350-499 cells/mm^3^  500-749 cells/mm^3^  ≥750 cells/mm^3^ | 22 (5.0%)  47 (10.7%)  149 (33.8%)  223 (50.6%) | -0.26 (-0.53 to +0.10)  -0.19 (-0.39 to +0.0004)  -0.13 (-0.26 to +0.0001)  REF | .045 | … ^C^ |  |
| **Current CD8 count ^D^**  <350 cells/mm^3^  350-499 cells/mm^3^  500-749 cells/mm^3^  ≥750 cells/mm^3^ | 110 (24.9%)  97 (22.0%)  92 (20.9%)  142 (32.2%) | REF  -0.07 (-0.24 to +0.10)  -0.004 (-0.18 to +0.17)  -0.14 (-0.29 to +0.02) | .271 | … ^C^ |  |

Values represent regression coefficients (β) of linear regression with 95% confidence interval. Unit is the log_10_ Median Fluorescence Intensity (MFI).

A. Number and percentage of participants for each variable category. B. At moment of post-vaccination blood draw. C. Variable not included in multivariable analysis. D. Last available data prior to receiving first vaccine dose of the primary vaccination course. E. Received one dose of ChAdOx1 and one dose of BNT162b2.

Abbreviations: BMI, body mass index; CI, confidence interval; P, p-value; REF, reference group.

**TABLE S5: Factors associated with SARS-CoV-2 anti-receptor binding domain IgG titres following vaccination against SARS-COV-2 in 195 people with HIV of the AGE_h_IV COVID-19 substudy.**

|  |  | **Univariable analysis** | | **Multivariable analysis** | |
| --- | --- | --- | --- | --- | --- |
|  | **n/195 (%) ^A^** | **β (95% CI)** | ***P*** | **β (95% CI)** | ***P*** |
| **Age ^B^**  <60 years  60-64 years  65-69 years  ≥70 years | 66 (33.9%)  49 (25.1%)  41 (21.0%)  39 (20.0%) | REF  -0.38 (-0.62 to -0.14)  -0.38 (-0.63 to -0.13)  -0.22 (-0.47 to +0.04) | .005 | … ^C^ |  |
| **Sex at birth**  Male  Female | 184 (94.4%)  11 (5.6%) | REF  +0.57 (+0.17 to +0.96) | .005 | REF  +0.42 (+0.10 to +0.75) | .010 |
| **Ethnic origin**  Caucasian  African  Asian | 190 (9.4%)  5 (2.6%)  0 (0.0%) | REF  +0.49 (-0.09 to +1.07)  - | .099 | … ^C^ |  |
| **BMI**, kg/m^2^ **^D^**  Underweight (<18.5)  Normal weight (18.5-24.9)  Overweight (25.0-29.9)  Obese (≥30.0) | 1 (0.5%)  101 (51.8%)  72 (36.9%)  21 (10.8%) | -0.17 (-1.46 to +1.12)  REF  +0.12 (-0.07 to +0.32)  -0.24 (-0.55 to +0.07) | .153 | -0.19 (-1.20 to +0.82)  REF  -0.06 (-0.22 to +0.10)  -0.35 (-0.59 to -0.11) | .041 |
| **Total comorbidities ^D^**  0 comorbidities  1-2 comorbidities  3-7 comorbidities | 79 (40.5%)  96 (49.2%)  20 (10.3%) | REF  -0.03 (-0.23 to +0.16)  -0.14 (-0.47 to +0.18) | .682 | … ^C^ |  |
| **Prior SARS-CoV-2 infection**  No  Yes | 167 (85.6%)  28 (14.4%) | REF  +0.70 (+0.45 to +0.94) | <.001 | REF  +0.73 (+0.53 to +0.94) | <.001 |
| **SARS-COV-2 vaccine**  mRNA-based  vector-based  heterologous ^E^ | 130 (66.7%)  64 (32.8%)  1 (0.5%) | +0.55 (+0.37 to +0.73)  REF  +0.36 (-0.84 to +1.57) | <.001 | +0.59 (+0.43 to +0.75)  REF  +0.56 (-0.45 to +1.57) | <.001 |
| **Days between last vaccine dose and post-vaccination sampling**  *(per 10-day increase)* |  | -0.08 (-0.13 to -0.03) | .002 | -0.06 (-0.10 to -0.02) | .004 |
| **Current CD4/8 ratio** **^D^**  < 0.50  0.50 – 0.99  ≥ 1.0 | 21 (10.8%)  98 (50.3%)  76 (39.0%) | -0.39 (-0.71 to -0.08)  -0.10 (-0.30 to +0.09)  REF | .052 | -0.45 (-0.70 to -0.20)  -0.11 (-0.27 to +0.05)  REF | .002 |
| **Current CD4 count ^D^**  <350 cells/mm^3^  350-499 cells/mm^3^  500-749 cells/mm^3^  ≥750 cells/mm^3^ | 19 (9.7%)  26 (13.3%)  77 (39.5%)  73 (37.5%) | -0.16 (-0.49 to +0.17)  -0.16 (-0.46 to +0.13)  -0.07 (-0.28 to +0.14)  REF | .638 | … ^C^ |  |
| **Current CD8 count ^D^**  <350 cells/mm^3^  350-499 cells/mm^3^  500-749 cells/mm^3^  ≥750 cells/mm^3^ | 22 (11.3%)  24 (12.3%)  50 (25.6%)  99 (50.8%) | REF  -0.10 (-0.48 to +0.28)  -0.02 (-0.35 to +0.32)  -0.13 (-0.44 to +0.17) | .703 | … ^C^ |  |
| **CD4nadir**  *(per 100 cells/mm^3^ increase)* |  | +0.02 (-0.04 to +0.07) | .574 | … ^C^ |  |
| **Years since HIV diagnosis** **^B^**  *(per 10 years increase)* |  | -0.09 (-0.21 to +0.04) | .181 | … ^C^ |  |
| **Years since starting ART ^B^**  *(per 10 years increase)* |  | -0.02 (-0.17 to +0.13) | .807 | … ^C^ |  |

Values represent regression coefficients (β) of linear regression with 95% confidence interval. Unit is the log_10_ Median Fluorescence Intensity (MFI).

A. Number and percentage of participants for each variable category. B. At moment of post-vaccination blood draw. C. Variable not included in multivariable analysis. D. Last available data prior to receiving first vaccine dose of the primary vaccination course. E. Received one dose of ChAdOx1 and one dose of BNT162b2.

Abbreviations: ART, antiretroviral therapy; BMI, body mass index; CI, confidence interval; P, p-value; REF, reference group.

**TABLE S6: Factors associated with SARS-CoV-2 anti-receptor binding domain IgG titers following vaccination against SARS-COV-2 in 246 controls without HIV of the AGE_h_IV COVID-19 substudy.**

|  |  | **Univariable analysis** | | **Multivariable analysis** | |
| --- | --- | --- | --- | --- | --- |
|  | **n/246 (%) ^A^** | **β (95% CI)** | ***P*** | **β (95% CI)** | ***P*** |
| **Age ^B^**  <60 years  60-64 years  65-69 years  ≥70 years | 101 (41.0%)  59 (24.0%)  40 (16.3%)  46 (18.7%) | REF  -0.19 (-0.38 to +0.001)  -0.08 (-0.30 to +0.13)  +0.14 (-0.07 to +0.34) | .037 | … ^C^ |  |
| **Sex at birth**  Male  Female | 211 (85.8%)  35 (14.2%) | REF  +0.18 (-0.03 to +0.39) | .095 | … ^C^ |  |
| **Ethnic origin**  Caucasian  African  Asian | 237 (96.4%)  4 (1.6%)  5 (2.0%) | REF  +0.32 (-0.27 to +0.91)  +0.29 (-0.24 to +0.81) | .329 | … ^C^ |  |
| **BMI**, kg/m^2^ **^D^**  Underweight (<18.5)  Normal weight (18.5-24.9)  Overweight (25.0-29.9)  Obese (≥30.0) | 0 (0.0%)  118 (48.0%)  97 (39.4%)  31 (12.6%) | -  REF  +0.07 (-0.09 to +0.23)  +0.16 (-0.07 to +0.40) | .370 | … ^C^ |  |
| **Total comorbidities ^D^**  0 comorbidities  1-2 comorbidities  3-7 comorbidities | 154 (62.6%)  78 (31.7%)  14 (5.7%) | REF  -0.01 (-0.18 to +0.15)  +0.08 (-0.25 to +0.41) | .867 | … ^C^ |  |
| **Prior SARS-CoV-2 infection**  No  Yes | 212 (86.2%)  34 (13.8%) | REF  +0.78 (+0.59 to +0.98) | <.001 | REF  +0.78 (+0.62 to +0.94) | <.001 |
| **SARS-COV-2 vaccine**  mRNA-based  vector-based  heterologous ^E^ | 173 (70.3%)  70 (28.4%)  3 (1.2%) | +0.53 (+0.38 to +0.68)  REF  +0.49 (-0.14 to +1.12) | <.001 | +0.38 (+0.25 to +0.50)  REF  +0.45 (-0.05 to +0.96) | <.001 |
| **Days between last vaccine dose and post-vaccination sampling**  *(per 10-day increase)* |  | -0.13 (-0.17 to -0.10) | <.001 | -0.11 (-0.14 to -0.08) | <.001 |
| **Current CD4/8 ratio** **^D^**  < 0.50  0.50 – 0.99  ≥ 1.0 | 0 (0.0%)  21 (8.5%)  225 (91.5%) | -  -0.30 (-0.56 to -0.03)  REF | .027 | -  -0.22 (-0.42 to -0.03)  REF | .025 |
| **Current CD4 count ^D^**  <350 cells/mm^3^  350-499 cells/mm^3^  500-749 cells/mm^3^  ≥750 cells/mm^3^ | 3 (1.2%)  21 (8.5%)  72 (29.3%)  150 (61.0%) | -0.62 (-1.30 to +0.05)  -0.20 (-0.47 to +0.07)  -0.16 (-0.33 to +0.002)  REF | .057 | … ^C^ |  |
| **Current CD8 count ^D^**  <350 cells/mm^3^  350-499 cells/mm^3^  500-749 cells/mm^3^  ≥750 cells/mm^3^ | 88 (35.8%)  73 (29.6%)  42 (17.1%)  43 (17.5%) | REF  -0.06 (-0.24 to +0.13)  +0.02 (-0.20 to +0.24)  -0.13 (-0.34 to +0.09) | .624 | … ^C^ |  |

Values represent regression coefficients (β) of linear regression with 95% confidence interval. Unit is the log_10_ Median Fluorescence Intensity (MFI).

A. Number and percentage of participants for each variable category. B. At moment of post-vaccination blood draw. C. Variable not included in multivariable analysis. D. Last available data prior to receiving first vaccine dose of the primary vaccination course. E. Received one dose of ChAdOx1 and one dose of BNT162b2.

Abbreviations: BMI, body mass index; CI, confidence interval; P, p-value; REF, reference group.

**TABLE S7: Factors associated with SARS-CoV-2 IFNγ-release following vaccination against SARS-COV-2 in 192 people with HIV of the AGE_h_IV COVID-19 substudy.**

|  |  | **Univariable analysis ^A^** | | **Multivariable analysis ^A^** | |
| --- | --- | --- | --- | --- | --- |
|  | **n/192 (%) ^B^** | **β (95% CI)** | ***P*** | **β (95% CI)** | ***P*** |
| **Age ^C^**  <60 years  60-64 years  65-69 years  ≥70 years | 64 (33.3%)  48 (25.0%)  41 (21.4%)  39 (20.3%) | REF  +0.08 (-0.99 to +1.15)  +0.32 (-0.81 to +1.45)  -0.94 (-2.16 to +0.28) | .238 | … ^D^ |  |
| **Sex at birth**  Male  Female | 181 (94.3%)  11 (5.7%) | REF  +0.59 (-1.13 to +2.30) | .499 | … ^D^ |  |
| **Ethnic origin**  Caucasian  African  Asian | 187 (97.4%)  5 (2.6%)  0 (0.0%) | REF  +1.19 (-1.27 to +3.64)  - | .342 | … ^D^ |  |
| **BMI**, kg/m^2^ **^E^**  Underweight (<18.5)  Normal weight (18.5-24.9)  Overweight (25.0-29.9)  Obese (≥30.0) | 1 (0.5%)  99 (51.6%)  72 (37.5%)  20 (10.4%) | -13.25 (-1032.34 to +1005.83)  REF  +0.04 (-0.83 to +0.90)  +0.23 (-1.13 to +1.59) | .990 | … ^D^ |  |
| **Total comorbidities ^E^**  0 comorbidities  1-2 comorbidities  3-7 comorbidities | 77 (40.1%)  95 (49.5%)  20 (10.4%) | REF  -0.27 (-1.12 to +0.58)  -1.36 (-2.82 to +0.11) | .190 | … ^D^ |  |
| **Prior SARS-CoV-2 infection**  No  Yes | 164 (85.4%)  28 (14.6%) | REF  +1.46 (+0.32 to +2.59) | .012 | REF  +1.42 (+0.31 to +2.53) | .013 |
| **SARS-COV-2 vaccine**  mRNA-based  vector-based  heterologous ^F^ | 129 (67.2%)  62 (32.3%)  1 (0.5%) | -0.18 (-1.04 to +0.68)  REF  +3.39 (-1.87 to +8.65) | .383 | -0.01 (-0.84 to +0.82)  REF  +3.87 (-1.19 to +8.93) | .316 |
| **Days between last vaccine dose and post-vaccination sampling**  *(per 10-day increase)* |  | -0.13 (-0.35 to +0.08) | .223 | … ^D^ |  |
| **Current CD4/8 ratio** **^E^**  < 0.50  0.50 – 0.99  ≥ 1.0 | 21 (10.9%)  97 (50.5%)  74 (38.6%) | -1.42 (-2.83 to -0.02)  -0.91 (-1.75 to -0.06)  REF | .044 | -1.39 (-2.76 to -0.02)  -0.90 (-1.72 to -0.07)  REF | .041 |
| **Current CD4 count ^E^**  <350 cells/mm^3^  350-499 cells/mm^3^  500-749 cells/mm^3^  ≥750 cells/mm^3^ | 19 (9.9%)  26 (13.5%)  76 (39.6%)  71 (37.0%) | -0.83 (-2.30 to +0.64)  -0.75 (-2.05 to +0.54)  -0.31 (-1.23 to +0.60)  REF | .557 | … ^D^ |  |
| **Current CD8 count ^E^**  <350 cells/mm^3^  350-499 cells/mm^3^  500-749 cells/mm^3^  ≥750 cells/mm^3^ | 21 (11.0%)  24 (12.5%)  49 (25.5%)  98 (51.0%) | REF  +0.004 (-1.61 to +1.62)  -0.47 (-1.89 to +0.95)  -1.05 (-2.37 to +0.26) | .199 | … ^D^ |  |
| **CD4nadir**  *(per 100 cells/mm^3^ increase)* |  | -0.02 (-0.26 to +0.22) | .863 | … ^D^ |  |
| **Years since HIV diagnosis** **^C^**  *(per 10 years increase)* |  | -0.12 (-0.68 to +0.43) | .662 | … ^D^ |  |
| **Years since starting ART ^C^**  *(per 10 years increase)* |  | +0.16 (-0.49 to +0.81) | .633 | … ^D^ |  |

Peripheral blood mononuclear cells (PBMC) for SARS-CoV-2 T-cell responses as measured by IFNγ-release assay were available from 192 of 195 people with HIV. Values represent regression coefficients (β) with 95% confidence interval of tobit regression (censored lower bound at 0.01pg/mL). Unit is the log_10_ pg/mL.

A. Univariable and multivariable analyses were adjusted for pre-vaccination level of IFNγ-release upon SARS-CoV-2 nucleocapsid- and spike-peptide pool stimulation. B. Number and percentage of participants for each variable category. C. At moment of post-vaccination blood draw. D. Variable not included in multivariable analysis. E. Last available data prior to receiving first vaccine dose of the primary vaccination course. F. Received one dose of ChAdOx1 and one dose of BNT162b2.

Abbreviations: ART, antiretroviral therapy; BMI, body mass index; CI, confidence interval; P, p-value; REF, reference group.

**TABLE S8: Factors associated with SARS-CoV-2 IFNγ-release following vaccination against SARS-COV-2 in 244 controls without HIV of the AGE_h_IV COVID-19 substudy.**

|  |  | **Univariable analysis ^A^** | | **Multivariable analysis ^A^** | |
| --- | --- | --- | --- | --- | --- |
|  | **n/244 (%) ^B^** | **β (95% CI)** | ***P*** | **β (95% CI)** | ***P*** |
| **Age ^C^**  <60 years  60-64 years  65-69 years  ≥70 years | 100 (41.0%)  58 (23.8%)  40 (16.4%)  46 (18.8%) | REF  +0.73 (-0.69 to +2.16)  -0.06 (-1.73 to +1.60)  -0.21 (-1.85 to +1.44) | .677 | … ^D^ |  |
| **Sex at birth**  Male  Female | 210 (86.1%)  34 (13.9%) | REF  +0.75 (-0.84 to +2.34) | .355 | … ^D^ |  |
| **Ethnic origin**  Caucasian  African  Asian | 235 (96.3%)  4 (1.6%)  2 (2.1%) | REF  +1.73 (-2.37 to +5.82)  +1.83 (-1.75 to +5.41) | .436 | … ^D^ |  |
| **BMI**, kg/m^2^ **^E^**  Underweight (<18.5)  Normal weight (18.5-24.9)  Overweight (25.0-29.9)  Obese (≥30.0) | 0 (0.0%)  116 (47.5%)  97 (39.8%)  31 (12.7%) | -  REF  -1.29 (-2.53 to -0.06)  -0.54 (-2.33 to +1.24) | .122 | … ^D^ |  |
| **Total comorbidities ^E^**  0 comorbidities  1-2 comorbidities  3-7 comorbidities | 153 (62.7%)  77 (31.6%)  14 (5.7%) | REF  -1.26 (-2.54 to +0.02)  +0.14 (-2.24 to +2.52) | .143 | … ^D^ |  |
| **Prior SARS-CoV-2 infection**  No  Yes | 211 (86.5%)  33 (13.5%) | REF  +3.40 (+1.88 to +4.91) | <.001 | REF  +3.41 (+1.96 to +4.86) | <.001 |
| **SARS-COV-2 vaccine**  mRNA-based  vector-based  heterologous ^F^ | 171 (70.1%)  70 (28.7%)  3 (1.2%) | -0.42 (-1.65 to +0.81)  REF  -15.92 (-818.50 to +786.66) | .800 | -0.79 (-1.98 to +0.39)  REF  -14.48 (-1247.25 to +1218.29) | .418 |
| **Days between last vaccine dose and post-vaccination sampling**  *(per 10-day increase)* |  | -0.28 (-0.58 to +0.01) | .061 | -0.37 (-0.66 to -0.08) | .013 |
| **Current CD4/8 ratio** **^E^**  < 0.50  0.50 – 0.99  ≥ 1.0 | 0 (0.0%)  21 (8.6%)  223 (91.4%) | -  +0.04 (-1.96 to +2.04)  REF | .968 | … ^D^ |  |
| **Current CD4 count ^E^**  <350 cells/mm^3^  350-499 cells/mm^3^  500-749 cells/mm^3^  ≥750 cells/mm^3^ | 3 (1.2%)  21 (8.6%)  72 (29.5%)  148 (60.7%) | +1.08 (-3.92 to +6.09)  +0.63 (-1.39 to +2.66)  +1.66 (+0.42 to +2.89)  REF | .074 | +2.02 (-2.60 to +6.64)  +0.62 (-1.28 to +2.52)  +1.70 (+0.55 to +2.85)  REF | .035 |
| **Current CD8 count ^E^**  <350 cells/mm^3^  350-499 cells/mm^3^  500-749 cells/mm^3^  ≥750 cells/mm^3^ | 88 (36.1%)  71 (29.1%)  42 (17.2%)  43 (17.6%) | REF  -1.34 (-2.75 to +0.06)  -1.46 (-3.13 to +0.22)  -1.02 (-2.63 to +0.59) | .179 | … ^D^ |  |

Peripheral blood mononuclear cells (PBMC) for SARS-CoV-2 T-cell responses as measured by IFNγ-release assay were available from 244 of 246 controls without HIV. Values represent regression coefficients (β) with 95% confidence interval of tobit regression (censored lower bound at 0.01pg/mL). Unit is the log_10_ pg/mL.

A. Univariable and multivariable analyses were adjusted for pre-vaccination level of IFNγ-release upon SARS-CoV-2 nucleocapsid- and spike-peptide pool stimulation. B. Number and percentage of participants for each variable category. C. At moment of post-vaccination blood draw. D. Variable not included in multivariable analysis. E. Last available data prior to receiving first vaccine dose of the primary vaccination course. F. Received one dose of ChAdOx1 and one dose of BNT162b2.

Abbreviations: BMI, body mass index; CI, confidence interval; P, p-value; REF, reference group

**TABLE S9: Immune phenotyping of T-cells and monocytes in 71 participants of the AGE_h_IV COVID-19 substudy (at 4 to 13 weeks after last dose of a SARS-CoV-2 vaccine), by HIV-status.**

|  | **People with HIV**  **(n = 36)** | **Controls**  **(n = 35)** | ***P*** |
| --- | --- | --- | --- |
| % Activated CD4-cells ^A^ | 0.40 (0.26 – 0.50) | 0.46 (0.26 – 0.58) | .325 |
| % Activated CD8-cells ^A^ | 0.86 (0.71 – 1.20) | 1.00 (0.70 – 1.39) | .700 |
| % Exhaustion CD4-cells ^A^ | 0.95 (0.84 – 1.12) | 0.94 (0.88 – 1.09) | .890 |
| % Senescence CD4-cells ^A^ | 0.70 (0.34 – 1.01) | 0.59 (-0.25 to +0.76) | .121 |
| % Exhaustion CD8-cells ^A^ | 1.18 (1.00 – 1.26) | 1.23 (0.99 – 1.34) | .059 |
| % Senescence CD8-cells ^A^ | 1.72 (1.63 – 1.78) | 1.63 (1.50 – 1.74) | .043 |
| % CD16 monocytes ^A^ | 7.92 (4.78 – 11.55) | 5.26 (3.42 – 7.86) | .018 |
| CD163 on classical monocytes ^B^ | 617 (546 – 705) | 535 (446 – 605) | **<**.**001** |
| CD64 on classical monocytes ^B^ | 1122 (935 – 1290) | 1048 (912 – 1171) | .095 |
| CD38 on classical monocytes ^B^ | 2595 (2372 – 2838) | 2514 (2192 – 2875) | .404 |
| CD32 on classical monocytes ^B^ | 6812 (4499 – 8614) | 7060 (5687 – 8549) | .469 |
| HLA-DR on classical monocytes ^B^ | 1651 (1440 – 2010) | 1594 (1362 – 1956) | .411 |
| CD163 on CD16+ monocytes ^B^ | 484 (417 – 553) | 543 (489 – 638) | .011 |
| CD64 on CD16+ monocytes ^B^ | 448 (398 – 609) | 552 (433 – 886) | .007 |
| CD38 on CD16+ monocytes ^B^ | 706 (575 – 856) | 885 (754 – 992) | **<**.**001** |
| CD32 on CD16+ monocytes ^B^ | 5267 (3803 – 6713) | 6196 (5302 – 7833) | .024 |
| HLA-DR on CD16+ monocytes ^B^ | 1901 (1395 – 2500) | 1781 (1522 – 2215) | .654 |

All values are median (interquartile range). Values were compared between people with HIV and controls using Wilcoxon rank-sum test. Uncorrected p values are given. Bonferroni adjustment for multiple testing required a p value of <0.003 for significance and are displayed in bold.

A. Values are log_10_ transformed to obtain normality. B. Unit is the geometric mean of fluorescence intensity.

**TABLE S10: Association of immune phenotyping with post-vaccination SARS-CoV-2-specific IFNγ-release in 71 participants of the AGE_h_IV COVID-19 substudy.**

|  | **Model 1 ^1^** | | **Model 2 ^2^** | |
| --- | --- | --- | --- | --- |
|  | **β (95% CI)** | **P** | **β (95% CI)** | **P** |
| Activated CD4-cells ^A^ | -2.75 (-7.50 to +2.00) | .252 | -1.99 (-6.65 to +2.66) | .395 |
| Activated CD8-cells ^A^ | +0.53 (-2.42 to +3.48) | .719 | +0.07 (-2.88 to +3.02) | .964 |
| Exhaustion CD4-cells ^A^ | -4.32 (-9.37 to +0.72) | .092 | -5.15 (-10.14 to -0.16) | .043 |
| Senescence CD4-cells ^A^ | -0.88 (-2.59 to +0.83) | .308 | -1.53 (-3.28 to +0.21) | .084 |
| Exhaustion CD8-cells ^A^ | -1.08 (-5.60 to +3.44) | .636 | -1.17 (-5.58 to +3.25) | .599 |
| Senescence CD8-cells ^A^ | -3.42 (-8.82 to +1.99) | .211 | -6.15 (-11.65 to -0.66) | .029 |
| CD16 monocytes ^A^ | +0.19 (-0.10 o +0.47) | .193 | +0.08 (-0.21 to +0.37) | .576 |
| CD163 on classical monocytes ^B^ | +2.30 (+1.16 to +3.44) | <.001 | +2.31 (+1.03 to +3.59) | .**001** |
| CD64 on classical monocytes ^B^ | +0.09 (-0.25 to +0.43) | .591 | +0.04 (-0.29 to +0.38) | .794 |
| CD38 on classical monocytes ^B^ | -0.13 (-0.41 to +0.16) | .371 | -0.19 (-0.48 to +0.09) | .179 |
| CD32 on classical monocytes ^B^ | +0.02 (-0.02 to +0.07) | .338 | +0.03 (-0.02 to +0.07) | .233 |
| HLA-DR on classical monocytes ^B^ | +0.11 (-0.14 to +0.36) | .385 | +0.10 (-0.15 to +0.35) | .430 |
| CD163 on CD16+ monocytes ^B^ | -1.19 (-2.41 to +0.04) | .057 | -0.94 (-2.20 to +0.32) | .140 |
| CD64 on CD16+ monocytes ^B^ | -0.78 (-1.44 to -0.13) | .020 | -0.65 (-1.31 to -0.0004) | .050 |
| CD38 on CD16+ monocytes ^B^ | -0.35 (-0.85 to +0.16) | .175 | -0.23 (-0.69 to +0.23) | .321 |
| CD32 on CD16+ monocytes ^B^ | -0.04 (-0.09 to +0.02) | .225 | -0.02 (-0.08 to +0.04) | .465 |
| HLA-DR on CD16+ monocytes ^B^ | -0.02 (-0.18 to +0.14) | .814 | -0.02 (-0.19 to +0.14) | .809 |

Values represent regression coefficients (β) with 95% confidence interval of tobit regression (censored lower bound at 0.01pg /mL). Unit is the log_10_ pg/mL IFNγ-release upon SARS-CoV-2 nucleocapsid- and spike-peptide pool stimulation. Uncorrected p values are given. Bonferroni adjustment for multiple testing required a p value of <0.003 for significance and are displayed in bold.

A. Coefficient per 1 increase in log_10_ (%). B. Coefficient per 100 increase in geometric mean of fluorescence intensity.

1 Model 1: adjusted for pre-vaccination level of IFNγ-release

2 Model 2: adjusted for pre-vaccination level of IFNγ-release, HIV-status, SARS-CoV-2 vaccine type and days between last vaccine dose and post-vaccination sampling.

**TABLE S11: Association of immune phenotyping with percentage reactive CD4+ T-cells in 71 participants of the AGE_h_IV COVID-19 substudy.**

|  | **Model 1 ^1^** | | | **Model 2 ^2^** | |
| --- | --- | --- | --- | --- | --- |
|  | **β (95% CI)** | **P** | **β (95% CI)** | | **P** |
| Activated CD4-cells ^A^ | +0.30 (-0.03 to +0.63) | .078 | +0.27 (-0.05 to +0.59) | | .096 |
| Activated CD8-cells ^A^ | +0.03 (-0.17 to +0.22) | .797 | +0.06 (-0.12 to +0.25) | | .501 |
| Exhaustion CD4-cells ^A^ | +0.28 (-0.03 to +0.59) | .077 | +0.42 (+0.12 to +0.72) | | .006 |
| Senescence CD4-cells ^A^ | +0.03 (-0.09 to +0.14) | .665 | +0.09 (-0.02 to +0.21) | | .121 |
| Exhaustion CD8-cells ^A^ | +0.22 (-0.08 to +0.53) | .150 | +0.23 (-0.06 to +0.52) | | .114 |
| Senescence CD8-cells ^A^ | +0.03 (-0.35 to +0.41) | .890 | +0.18 (-0.20 to +0.56) | | .358 |
| CD16 monocytes ^A^ | -0.009 (-0.03 to +0.01) | .354 | -0.003 (-0.02 to +0.02) | | .771 |
| CD163 on classical monocytes ^B^ | -0.17 (-0.23 to -0.10) | <.001 | -0.16 (-0.23 to -0.09) | | **<**.**001** |
| CD64 on classical monocytes ^B^ | -0.001 (-0.02 to +0.02) | .934 | +0.003 (-0.02 to +0.02) | | .825 |
| CD38 on classical monocytes ^B^ | -0.01 (-0.03 to +0.005) | .140 | -0.01 (-0.03 to +0.009) | | .302 |
| CD32 on classical monocytes ^B^ | +0.00003 (-0.003 to +0.003) | .984 | -0.0005 (-0.004 to +0.003) | | .748 |
| HLA-DR on classical monocytes ^B^ | -0.01 (-0.03 to +0.004) | .123 | -0.01 (-0.03 to +0.004) | | .135 |
| CD163 on CD16+ monocytes ^B^ | +0.14 (+0.07 to +0.21) | <.001 | +0.13 (+0.05 to +0.20) | | .**001** |
| CD64 on CD16+ monocytes ^B^ | +0.05 (+0.03 to +0.07) | <.001 | +0.05 (+0.02 to +0.07) | | **<**.**001** |
| CD38 on CD16+ monocytes ^B^ | +0.02 (+0.0007 to +0.04) | .042 | +0.02 (-0.003 to +0.04) | | .097 |
| CD32 on CD16+ monocytes ^B^ | +0.004 (+0.0001 to +0.008) | .041 | +0.003 (-0.001 to +0.006) | | .167 |
| HLA-DR on CD16+ monocytes ^B^ | +0.0006 (-0.01 to +0.01) | .918 | -0.0007 (-0.01 to +0.01) | | .897 |

Values represent regression coefficients (β) and their 95% confidence interval of linear regression with a random intercept to account for variation between pairs. Unit is log_10_ percentage reactive CD4+ T-cells. Uncorrected p values are given. Bonferroni adjustment for multiple testing required a p value of <0.003 for significance and are displayed in bold.

A. Coefficient per 1 increase in log_10_ (%). B. Coefficient per 100 increase in geometric mean of fluorescence intensity.

1 Model 1: unadjusted

2 Model 2: adjusted for HIV-status, SARS-CoV-2 vaccine type and days between last vaccine dose and post-vaccination sampling.

**TABLE S12: Association of immune phenotyping with percentage reactive CD8+ T-cells in 71 participants of the AGE_h_IV COVID-19 substudy.**

|  | **Model 1 ^1^** | | **Model 2 ^2^** | |
| --- | --- | --- | --- | --- |
|  | **β (95% CI)** | **P** | **β (95% CI)** | **P** |
| Activated CD4-cells ^A^ | -0.04 (-0.44 to +0.35) | .831 | -0.09 (-0.43 to +0.25) | .604 |
| Activated CD8-cells ^A^ | -0.03 (-0.25 to +0.20) | .815 | +0.07 (-0.13 to +0.27) | .493 |
| Exhaustion CD4-cells ^A^ | -0.47 (-0.83 to -0.11) | .010 | -0.29 (-0.61 to +0.04) | .085 |
| Senescence CD4-cells ^A^ | -0.09 (-0.23 to +0.04) | .174 | -0.01 (-0.13 to +0.11) | .871 |
| Exhaustion CD8-cells ^A^ | -0.15 (-0.52 to +0.21) | .409 | -0.14 (-0.45 to +0.17) | .370 |
| Senescence CD8-cells ^A^ | -0.03 (-0.47 to +0.42) | .901 | +0.07 (-0.33 to +0.47) | .737 |
| CD16 monocytes ^A^ | -0.001 (-0.02 to +0.02) | .922 | +0.002 (-0.02 to +0.02) | .875 |
| CD163 on classical monocytes ^B^ | -0.12 (-0.20 to -0.03) | .007 | -0.10 (-0.18 to -0.02) | .016 |
| CD64 on classical monocytes ^B^ | +0.01 (-0.02 to +0.04) | .441 | +0.01 (-0.008 to +0.04) | .208 |
| CD38 on classical monocytes ^B^ | -0.04 (-0.06 to -0.01) | .001 | -0.03 (-0.05 to -0.01) | .**003** |
| CD32 on classical monocytes ^B^ | +0.004 (+0.00 to +0.008) | .050 | +0.003 (-0.0003 to +0.006) | .076 |
| HLA-DR on classical monocytes ^B^ | -0.02 (-0.04 to -0.005) | .012 | -0.03 (-0.04 to -0.01) | .**001** |
| CD163 on CD16+ monocytes ^B^ | +0.11 (+0.02 to +0.19) | .015 | +0.06 (-0.02 to +0.14) | .164 |
| CD64 on CD16+ monocytes ^B^ | +0.05 (+0.02 to +0.08) | .001 | +0.04 (+0.01 to +0.07) | .**003** |
| CD38 on CD16+ monocytes ^B^ | +0.02 (-0.007 to +0.04) | .183 | +0.01 (-0.006 to +0.04) | .170 |
| CD32 on CD16+ monocytes ^B^ | +0.006 (+0.002 to +0.01) | .003 | +0.004 (+0.0005 to +0.008) | .025 |
| HLA-DR on CD16+ monocytes ^B^ | -0.006 (-0.02 to +0.007) | .370 | -0.01 (-0.02 to -0.002) | .019 |

Values represent regression coefficients (β) and their 95% confidence interval of linear regression with a random intercept to account for variation between pairs. Unit is log_10_ percentage reactive CD8+ T-cells. Uncorrected p values are given. Bonferroni adjustment for multiple testing required a p value of <0.003 for significance and are displayed in bold.

A. Coefficient per 1 increase in log_10_ (%). B. Coefficient per 100 increase in geometric mean of fluorescence intensity.

1 Model 1: unadjusted

2 Model 2: adjusted for HIV-status, SARS-CoV-2 vaccine type and days between last vaccine dose and post-vaccination sampling.

**TABLE S13: Association of immune phenotyping with post-vaccination SARS-CoV-2 anti-spike IgG titers in 71 participants of the AGE_h_IV COVID-19 substudy.**

|  | **Model 1 ^1^** | | **Model 2 ^2^** | |
| --- | --- | --- | --- | --- |
|  | **β (95% CI)** | **P** | **β (95% CI)** | **P** |
| Activated CD4-cells ^A^ | -0.08 (-0.69 to +0.52) | .794 | +0.02 (-0.50 to +0.53) | .954 |
| Activated CD8-cells ^A^ | -0.20 (-0.54 to +0.14) | .249 | +0.08 (-0.23 to +0.38) | .625 |
| Exhaustion CD4-cells ^A^ | -0.65 (-1.22 to -0.07) | .027 | -0.24 (-0.76 to +0.28) | .363 |
| Senescence CD4-cells ^A^ | -0.15 (-0.35 to +0.05) | .148 | -0.05 (-0.24 to +0.13) | .574 |
| Exhaustion CD8-cells ^A^ | -0.27 (-0.86 to +0.32) | .362 | -0.04 (-0.57 to +0.49) | .881 |
| Senescence CD8-cells ^A^ | -0.25 (-0.91 to +0.41) | .461 | +0.01 (-0.61 to +0.64) | .968 |
| CD16 monocytes ^A^ | +0.02 (-0.02 to +0.05) | .280 | +0.02 (-0.01 to +0.05) | .185 |
| CD163 on classical monocytes ^B^ | +0.06 (-0.07 to +0.20) | .376 | +0.09 (-0.04 to +0.22) | .182 |
| CD64 on classical monocytes ^B^ | -0.02 (-0.06 to +0.03) | .464 | -0.01 (-0.05 to +0.03) | .600 |
| CD38 on classical monocytes ^B^ | -0.03 (-0.07 to +0.002) | .066 | -0.02 (-0.05 to +0.01) | .239 |
| CD32 on classical monocytes ^B^ | -0.002 (-0.007 to +0.004) | .570 | -0.003 (-0.008 to +0.002) | .299 |
| HLA-DR on classical monocytes ^B^ | +0.009 (-0.02 to +0.04) | .544 | +0.02 (-0.008 to +0.05) | .171 |
| CD163 on CD16+ monocytes ^B^ | -0.02 (-0.15 to +0.11) | .747 | -0.03 (-0.15 to +0.10) | .674 |
| CD64 on CD16+ monocytes ^B^ | -0.01 (-0.06 to +0.03) | .508 | -0.01 (-0.06 to +0.03) | .501 |
| CD38 on CD16+ monocytes ^B^ | -0.007 (-0.04 to +0.03) | .696 | +0.01 (-0.02 to +0.04) | .530 |
| CD32 on CD16+ monocytes ^B^ | -0.003 (-0.009 to +0.004) | .401 | -0.004 (-0.01 to +0.001) | .138 |
| HLA-DR on CD16+ monocytes ^B^ | +0.01 (-0.008 to +0.03) | .277 | +0.007 (-0.01 to +0.02) | .393 |

Values represent regression coefficients (β) and their 95% confidence interval of linear regression with a random intercept to account for variation between pairs. Unit is the log_10_ Median Fluorescence Intensity (MFI). Uncorrected p values are given. Bonferroni adjustment for multiple testing required a p value of <0.003 for significance and are displayed in bold.

A. Coefficient per 1 increase in log_10_ (%). B. Coefficient per 100 increase in geometric mean of fluorescence intensity.

1 Model 1: unadjusted

2 Model 2: adjusted for HIV-status, sex at birth, SARS-CoV-2 vaccine type and days between last vaccine dose and post-vaccination sampling.

**Table S14: Association of immune phenotyping with post-vaccination neutralization against ancestral SARS-CoV-2 in 71 participants of the AGE_h_IV COVID-19 substudy.**

|  | **Model 1 ^1^** | | **Model 2 ^2^** | |
| --- | --- | --- | --- | --- |
|  | **β (95% CI)** | **P** | **β (95% CI)** | **P** |
| Activated CD4-cells ^A^ | +0.18 (-0.29 to +0.64) | .463 | +0.22 (-0.19 to +0.63) | .292 |
| Activated CD8-cells ^A^ | +0.19 (-0.07 to +0.46) | .159 | +0.22 (-0.02 to +0.46) | .074 |
| Exhaustion CD4-cells ^A^ | -0.30 (-0.74 to +0.14) | .178 | -0.02 (-0.43 to +0.39) | .923 |
| Senescence CD4-cells ^A^ | -0.03 (-0.19 to +0.14) | .751 | +0.04 (-0.11 to +0.20) | .570 |
| Exhaustion CD8-cells ^A^ | +0.01 (-0.42 to +0.45) | .963 | +0.20 (-0.18 to +0.58) | .300 |
| Senescence CD8-cells ^A^ | -0.004 (-0.53 to +0.53) | .988 | +0.20 (-0.28 to +0.69) | .408 |
| CD16 monocytes ^A^ | +0.009 (-0.02 to +0.04) | .492 | +0.01 (-0.01 to +0.04) | .339 |
| CD163 on classical monocytes ^B^ | +0.01 (-0.10 to +0.12) | .856 | +0.04 (-0.06 to +0.13) | .436 |
| CD64 on classical monocytes ^B^ | +0.02 (-0.01 to +0.05) | .270 | +0.02 (-0.006 to +0.05) | .129 |
| CD38 on classical monocytes ^B^ | -0.01 (-0.04 to +0.01) | .304 | +0.003 (-0.02 to +0.03) | .802 |
| CD32 on classical monocytes ^B^ | +0.001 (-0.004 to +0.005) | .736 | -0.001 (-0.005 to +0.003) | .558 |
| HLA-DR on classical monocytes ^B^ | +0.02 (-0.004 to +0.04) | .096 | +0.02 (-0.004 to +0.04) | .115 |
| CD163 on CD16+ monocytes ^B^ | +0.02 (-0.09 to +0.13) | .723 | +0.01 (-0.08 to +0.10) | .834 |
| CD64 on CD16+ monocytes ^B^ | +0.009 (-0.03 to +0.04) | .643 | +0.01 (-0.02 to +0.05) | .404 |
| CD38 on CD16+ monocytes ^B^ | +0.02 (-0.008 to +0.05) | .154 | +0.03 (+0.007 to +0.05) | .011 |
| CD32 on CD16+ monocytes ^B^ | -0.0009 (-0.006 to +0.004) | .746 | -0.003 (-0.007 to +0.002) | .224 |
| HLA-DR on CD16+ monocytes ^B^ | +0.02 (+0.001 to +0.03) | .032 | +0.009 (-0.005 to +0.02) | .193 |

Values represent regression coefficients (β) and their 95% confidence interval of linear regression with a random intercept to account for variation between pairs. Unit is the log_10_ of serum dilution at which 50% of the infectivity was inhibited (ID50). Uncorrected p values are given. Bonferroni adjustment for multiple testing required a p value of <0.003 for significance and are displayed in bold.

A. Coefficient per 1 increase in log_10_ (%). B. Coefficient per 100 increase in geometric mean of fluorescence intensity.

1 Model 1: unadjusted

2 Model 2: adjusted for HIV-status, age, SARS-CoV-2 vaccine type and days between last vaccine dose and post-vaccination sampling.

**FIGURE S2: Schematic overview of the AGE_h_IV Cohort Study visits and the additional COVID-19 substudy visits.**


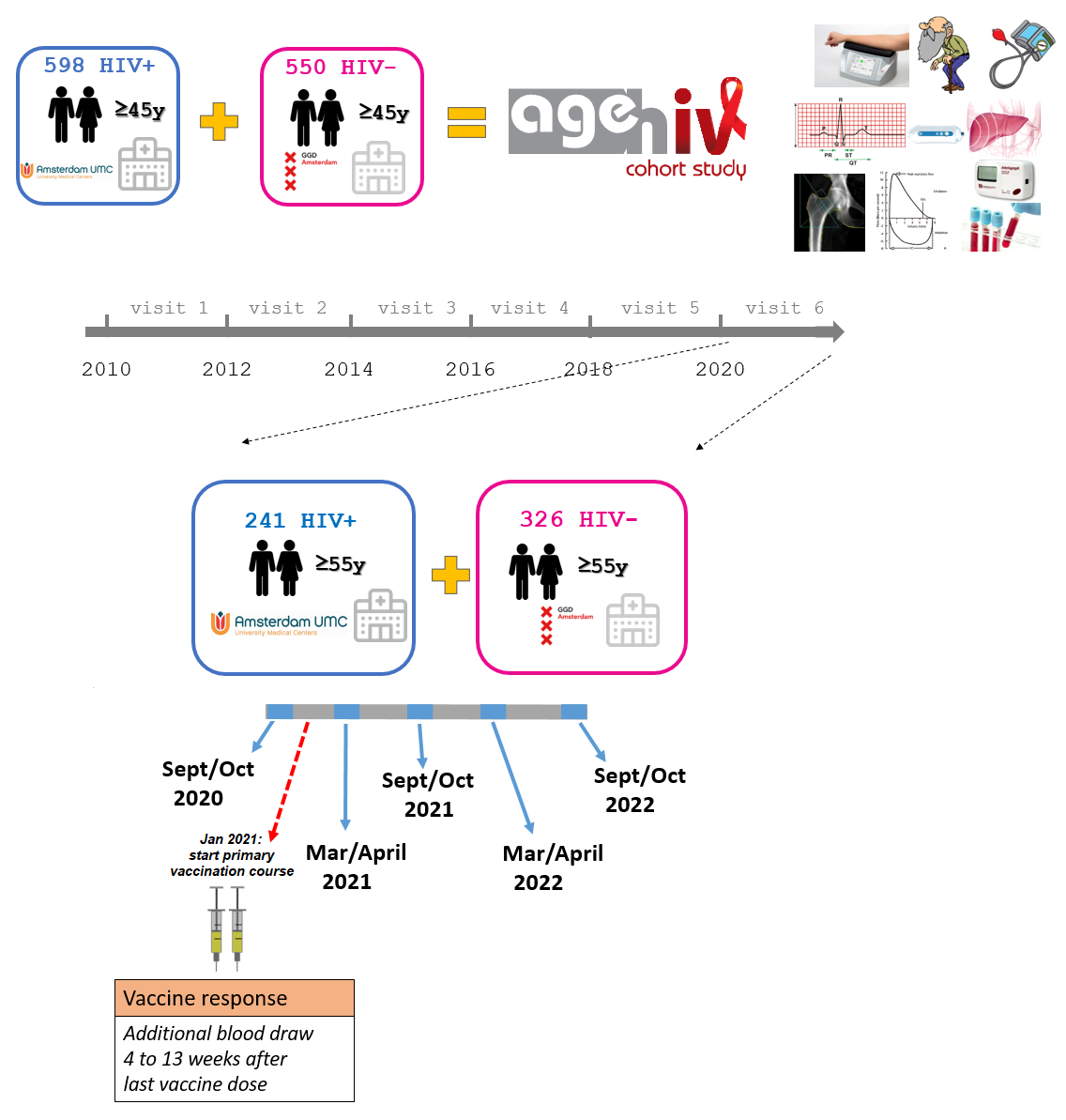


The AGE_h_IV Cohort Study is a prospective observational cohort study in people with and without HIV who were 45 years or older at the time of recruitment into the cohort. Between 2010 and 2012, PWH were recruited at the outpatient HIV-clinic of the Amsterdam University Medical Centers (Amsterdam UMC), location AMC, and controls from the sexual health clinic and the Amsterdam Cohort Studies on HIV/AIDS at the Public Health Service Amsterdam. Every two years, patients undergo screening for age-related comorbidities.

In September 2020, a COVID-19 substudy, encompassing five six-monthly study visits between September 2020 and October 2022, started including AGE_h_IV Cohort participants in active follow-up and residing in the Netherlands. In addition to these five substudy visits, participants were invited for an additional blood draw 4 to 13 weeks after completing primary SARS-CoV-2 vaccination. The Dutch national COVID-19 vaccination program started in January 2021.

**TEXT S1: Supplementary text to ‘Methods’ section**

**Study design and participants**

The AGE_h_IV Cohort Study is a prospective observational cohort study assessing the prevalence and incidence of age-related comorbidities and their risk factors in people 45 years or older with and without HIV.^1^ Between 2010 and 2012, PWH were recruited at the outpatient HIV-clinic of the Amsterdam University Medical Centers (Amsterdam UMC), location AMC, and controls from the sexual health clinic and the Amsterdam Cohort Studies on HIV/AIDS at the Public Health Service Amsterdam, resulting in a control group with highly similar socio-demographic and behavioural characteristics. At baseline and every 2 years thereafter, patients undergo standardized screening for age-related comorbidities, and collection of blood, urine and stool for cryopreservation.

In August 2020, following the first SARS-CoV-2 epidemic wave in the Netherlands, all AGE_h_IV Cohort participants in active follow-up and residing in the Netherlands were asked to participate in a COVID-19 substudy, encompassing five six-monthly study visits between September 2020 and October 2022.^2^ During each visit, blood samples are obtained and participants complete a standardized study questionnaire.

In addition to these five study visits, participants were invited for an additional blood draw 4 to 13 weeks after completing primary vaccination with BNT162b2, mRNA-1273, ChAdOx1 (two injections 4 to 12 weeks apart or a single injection for those with documented prior SARS-CoV-2 infection) or Ad26.COV2.S (single injection) according to Dutch vaccination guidelines. For the current analysis, data collected between September 2020 (first six-monthly COVID-19 substudy visit) until November 2021 (last additional blood draw after completing primary vaccination) were used. The blood sample most closely preceding the first dose of a SARS-CoV-2 vaccine (pre-vaccination) and the blood sample drawn 4-13 weeks after the last dose (post-vaccination) were analysed.

**Prior SARS-CoV-2 infection**

SARS-CoV-2 nucleocapsid (N)-antibody levels were measured to determine prior SARS-CoV-2 infection, using the semi-quantitative INgezim® COVID-19 double recognition (DR) assay (Eurofins Ingenasa, Madrid, Spain), which captures the combined IgA/IgM/IgG antibody response to the SARS-CoV-2 N-protein (sensitivity 100%, specificity 98.2%^3^). N-antibody levels were expressed as a ratio of the sample to positive control (S/Co) for each sample, which was calculated as *((optical density (OD) sample-OD blank) / (OD positive control-OD blank)) x 10*. In accordance with the manufacturer’s instructions an S/Co ratio ≥6 was considered to represent a positive SARS-CoV-2 N-antibody response, and used to define prior SARS-CoV-2 infection.

Participants were considered to have a prior SARS-CoV-2 infection in case of a self-reported positive PCR-test and/or a positive SARS-CoV-2 nucleocapsid (N)-antibody test.

**References**

1 Schouten, J. *et al.* Cross-sectional comparison of the prevalence of age-associated comorbidities and their risk factors between HIV-infected and uninfected individuals: the AGEhIV cohort study. *Clin Infect Dis* **59**, 1787-1797, doi:10.1093/cid/ciu701 (2014).

2 Verburgh, M. L. *et al.* Similar Risk of Severe Acute Respiratory Syndrome Coronavirus 2 Infection and Similar Nucleocapsid Antibody Levels in People With Well-Controlled Human Immunodeficiency Virus (HIV) and a Comparable Cohort of People Without HIV. *J Infect Dis* **225**, 1937-1947, doi:10.1093/infdis/jiab616 (2022).

3 Hoste, A. C. R. *et al.* Two serological approaches for detection of antibodies to SARS-CoV-2 in different scenarios: a screening tool and a point-of-care test. *Diagn Microbiol Infect Dis* **98**, 115167, doi:10.1016/j.diagmicrobio.2020.115167 (2020).

**FIGURE S3: Overview of measurements in 441 included participants in the AGE_h_IV COVID-19 substudy.**


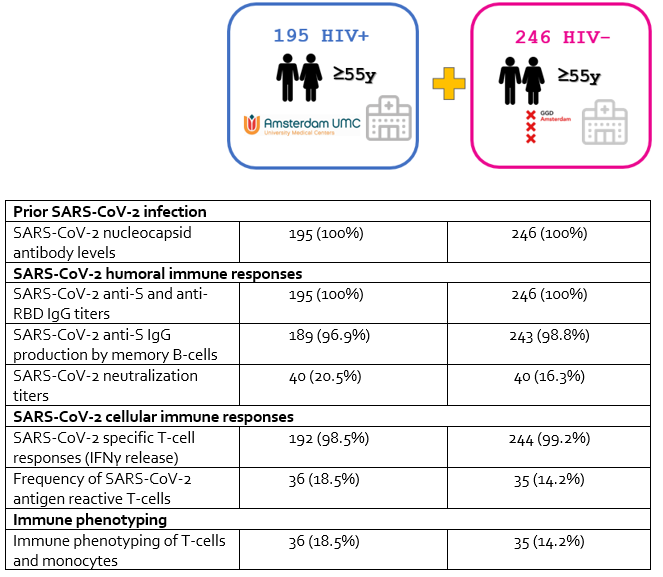


In 441 participants (195 PWH and 246 controls), pre- and post-vaccination blood samples were available.

- In all participants, SARS-CoV-2 nucleocapsid antibody levels and SARS-CoV-2 anti-spike(S) and anti-receptor binding domain(RBD) IgG titers were measured.

- In 436 of 441 participants, peripheral blood mononuclear cells (PBMC) were available. In 432 of 436, SARS-CoV-2 anti-S IgG production by memory B-cells was measured.

- A random selection of 40 people with HIV were matched 1:1 on age, sex and vaccine type to 40 controls. In these 80 participants, SARS-CoV-2 neutralization against SARS-CoV-2 ancestral, Delta and Omicron BA.1 variants was determined.

- SARS-CoV-2-specific T-cell responses were measured by IFNγ-release assay in 436 participants with available PBMC.

- In 71 of the 80 abovementioned subgroup participants with available PBMC, the percentage of SARS-CoV-2 antigen reactive T-cells was determined.

- In the same 71 participants, immune phenotyping of T-cells and monocytes was performed.
